# First detection and molecular identification of *Babesia* sp*.* from the giant panda, *Ailuropoda melanoleuca*, in China

**DOI:** 10.1186/s13071-020-04412-8

**Published:** 2020-10-29

**Authors:** Chanjuan Yue, Zeshuai Deng, Dunwu Qi, Yunli Li, Wenlei Bi, Rui Ma, Guangyou Yang, Xue Luo, Rong Hou, Songrui Liu

**Affiliations:** 1 Sichuan Key Laboratory of Conservation Biology for Endangered Wildlife, Chengdu Research Base of Giant Panda Breeding, Sichuan Academy of Giant Panda, 1375 Panda Road, Chenghua District, 610081 Sichuan China; 2 College of Veterinary Medicine, Sichuan Agricultural University, Chengdu, 611130 China

**Keywords:** *Babesia*, Giant panda, ailuropoda melanoleuca, Phylogenetic tree, *18S* rRNA gene

## Abstract

**Background:**

Parasitic infections are among the important causes of death of giant pandas (*Ailuropoda melanoleuca*) that hamper their survival in the wild. There are about 35 species of parasites which have been identified in giant pandas, but no information is currently available regarding the infection of *Babesia* in giant pandas. *Babesia* spp. are common intraerythrocytic parasite in wildlife, transmitted by ixodid ticks, which cause babesiosis. Clinical signs of babesiosis include fever, hemolysis, anemia, jaundice and death.

**Methods:**

A species of *Babesia* was detected in the blood of a giant panda based on morphology and PCR amplification of the *18S* rRNA gene. The phylogenetic relationship of *Babesia* sp. infecting giant panda was assessed by gene sequence alignment and phylogenetic analysis.

**Results:**

Our analysis revealed that the *Babesia* isolate detected was most similar to an unidentified species of *Babesia* identified in black bears (*Ursus thibetanus japonicus*) from Japan (*Babesia* sp*.* Iwate*,* AB586027.1) with a 99.56% sequence similarity, followed by *Babesia* sp*. EBB* (AB566229.1, 99.50%) and *Babesia* sp. Akita (AB566229.1, 99.07%).

**Conclusions:**

To our knowledge, this is the first report of *Babesia* detected in the giant panda. The results indicate that this *Babesia* sp. may be a novel species, currently named *Babesia* sp*.* strain EBP01.
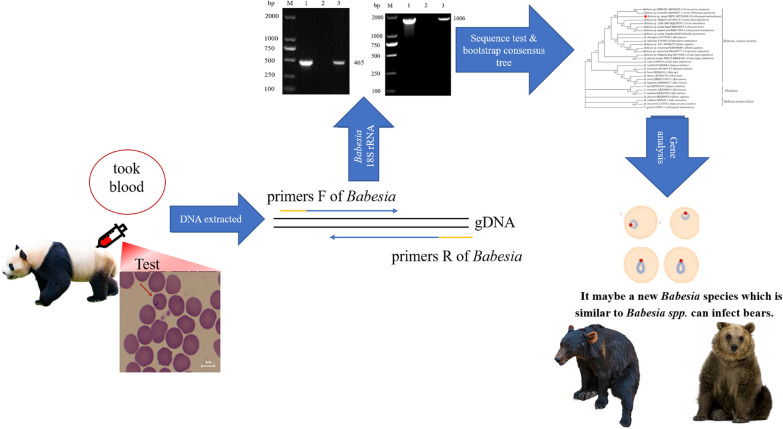

## Background

*Babesia* spp. are intraerythrocytic apicomplexan protozoan parasites known to infect wild and domestic animals and humans [1]. With a near global distribution, over 120 species of *Babesia* have been identified, most of which are transmitted by ixodid ticks [2]. These protozoan parasites are not strictly host-specific and this has contributed to the growing list of new species, with several *Babesia* species having been found to infect both animals and humans. Most cases of human babesiosis are caused by *B. microti*, *B. divergens*, *B. venatorum* and *B. duncani* and several unnamed species [3]. Geographically, *Babesia* spp. are widely distributed on all continents except Antarctica, especially in tropical and subtropical temperate zones.

The clinical presentation of a *Babesia* infection is termed ‘babesiosis,’ with signs consistent with parasite entry into and destruction of erythrocytes. Typical clinical signs of babesiosis include fever, hemolysis, anemia, jaundice and even MODS [4]. Babesiosis may be clinically characterized as asymptomatic to severe morbidity and death. Whether the host will be infected and suffer clinical disease also depends on the specific species [5–7]. Susceptibility to a *Babesia* infection often depends on the age and immune resistance of the host; older and low-immunity hosts are more susceptible to infection [8]. It seems that infection with *B. microti* in elderly, immunosuppressed and splenectomized patients have more severe and protracted illness [9].

Various *Babesia* species have been sporadically identified in wildlife, while the existence of the chronic asymptomatic carrier state in babesial infections of domestic and wild animals has been recognized for many years [10, 11]; as a result, babesiosis in wildlife has not received much attention. Despite ixodid ticks are implicated as the primary vector for *Babesia* spp., wildlife species are considered to be the reservoir, with several recent cases of fatal babesiosis were recorded in wildlife [12–14]. There remains a paucity in the literature for what species may be affected and what factors may be caused the clinical signs, and therefore, babesiosis in wildlife deserves further attention.

As an iconic “flagship” species for wildlife conservation, the giant panda (*Ailuropoda melanoleuca*) is considered a national treasure with the highest legal protection afforded by China. The giant panda is currently categorized as vulnerable by the IUCN, with both the *in-situ* and *ex-situ* populations facing continued threats from low reproductive rates, parasitic infection and infectious diseases [15, 16]. Regarding parasitic infection of giant pandas, previous research has focused on intestinal parasites, however, no information is currently available regarding the infection and genetic characteristics of *Babesia* in the giant panda [17].

The objective of the present study is to determine the phylogentic relationships, as well as the genetic and morphological characteristics of a *Babesia* species detected in a giant panda suffering from clinical babesiosis.

## Methods

An intraerythrocytic hemoparasite was detected on a blood smear from one female giant panda with the associated signs of fever, anemia, jaundice and hemoglobinuria. The blood smear demonstrated intraerythrocytic parasites with a morphology similar to that of known *Babesia* species, then the pathogen investigation was carried out by the following methods.

### Blood samples

Blood samples were collected from the basilica vein of the right or left arm of the giant panda without anesthesia, using a vacutainer containing EDTA, and then stored at 4 °C until DNA extraction.

### Detecting of *Babesia* in thin blood smears

Morphological observation of *Babesia* parasites was carried out under light microscopy. Thin blood smears were prepared, fixed with methanol (100% or absolute) and dried completely before staining. Two different stains, Giemsa (Sigma-Aldrich, Burlington, USA) and Diff Quick (Labokine™, Bad Kissingen, Germany), were performed according to the manufacturerʼs instructions. The slides were examined by light microscope at ×1000 under an oil immersion lens [18].

### DNA extraction

DNA was extracted from 200 μl of whole blood with the QIAamp 96 DNA Blood Kit (Qiagen, Hilden, Germany). DNA was eluted in 100 μl of elution buffer and used for identification of *Babesia*. According to the manufacturer’s instructions, the concentration of DNA was determined with a NanoDrop 2,000 spectrophotometer (NanoDrop Technologies, Wilmington, DE, USA). The DNA was stored at – 20 °C until further analysis.

### Detection of* Babesia *spp*.* by PCR

*Babesia* sp. was identified by using PCR with pfu DNA polymerase and primary primers F1 (5ʹ-GTC TTG TAA TTG GAA TGA TGG-3ʹ) and R1 (5ʹ-TAG TTT ATG GTT AGG ACT ACG-3ʹ) [19]. Amplification consisted of an initial denaturing at 95 °C for 5 min, followed by 40 cycles of 95 °C for 30 s, 56 °C for 30 s and 72 °C for 30 s, with a final extension step at 72 °C for 10 min. The expected size of amplicons is 465 bp which is a partial sequence of *Babesia 18S* rRNA gene. The targeted gene was amplified from template in 25 μl PCR mixtures containing 12.5 μl of PCR mix (Sangon Biotech^®^), 2 μl of template DNA , and 1 μl each of primer F1 and R1. The amplified DNA fragment was visualized with SYBR^®^ Safe (Thermo Fisher Scientific, Waltham, USA) after electrophoresis in 2 % agarose gel, then cloned and sequenced by Sangon Biotech^®^ (Shanghai, China). In addition, *18S* rRNA synthetic plasmid of the sequence of *B. canis* (GenBank: KT008057.1) and distilled water were used as positive and negative controls, respectively.

### Amplification and sequencing of nearly full length of *Babesia 18S* rRNA gene

The primer used with the Primer 5 (PREMIER Biosoft, Vancouver, BC, Canada) software was designed and the *Babesia* sequences came from NCBI database. The primers 18SF (5ʹ-TCC TGC CAG TAG TCA TA-3ʹ) and 18SR (5ʹ-TTG TTA CGA CTT CTC CT-3ʹ) were designed to amplify approximately 1600 bp of *Babesia 18S* rRNA gene sequence. According to the manufacturerʼs instructions, PCR was carried out in 25 μl reaction mixture comprised of 0.5 μl of dNTP (Thermo Fisher^®^), 2.5 μl of SuperFi 10X High Fidelity PCR Buffer, 0.1 μl of Plstinum Taq DNA polymerase High Fidelity (5U/μl, Thermo Fisher Scientific), 2 μl of DNA, 1 μl each of primer 18SF and 18SR, 1 μl of 50 mM MgSO_4_. Amplification consisted of an initial denaturing at 95 °C for 5 min, followed by 35 cycles of 95 °C for 60 s, 52 °C for 60 s and 72 °C for 60 s, with a final extension step at 72 °C for 10 min. The negative and positive controls were the same as described above. Amplified products were visualized with SYBR® Safe (Thermo Fisher Scientific) after electrophoresis in 2% agarose gel, then cloned and sequenced by Sangon Biotech^®^ (Shanghai, China).

### Sequence analysis

The obtained sequences were aligned with full length sequences of *18S* rRNA gene for related *Babesia* spp*.* by MegAlign component of the DNAStar software program. A phylogenetic analysis of the *18S* rRNA gene was performed by the neighbour-joining (NJ) method to estimate the phylogeny of the entire alignment using MEGA 7.0 software [2]. For this study, all 29 sequences for the other species were obtained from GenBank [20]. The phylogenetic tree was constructed and performed with 1000 bootstrap replications to evaluate the reliability of the constructions [21]. The distance matrices for the aligned sequences were calculated by the Kimura 2-parameter method, and there were a total of 1102 positions in the final dataset.

## Results

### Morphological observation

**Fig. 1:**
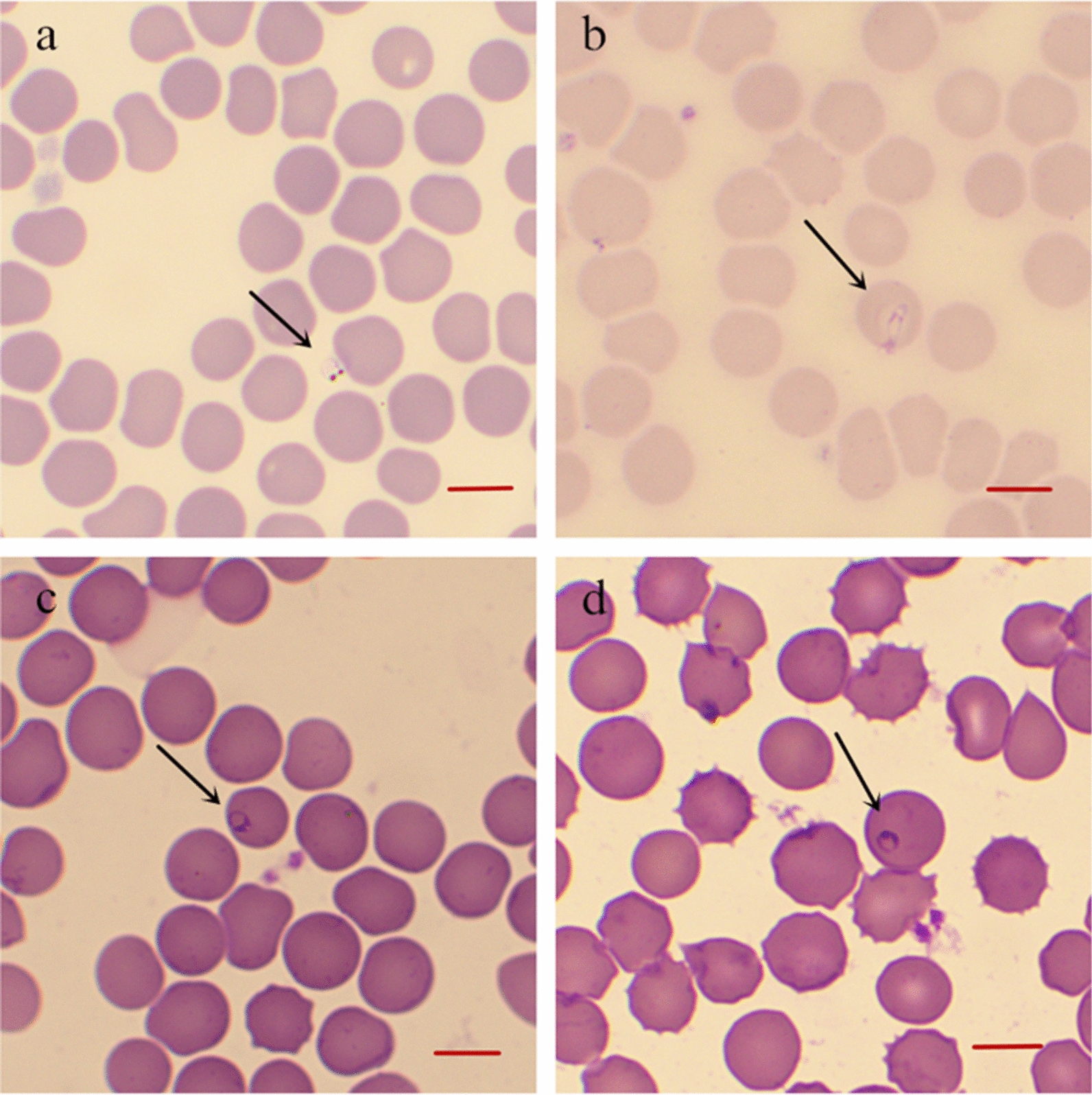
**a**, **b** Giemsa stained thin blood smear of *Babesia* from the giant panda. Final magnification of 1000×. **c**, **d** Diff Quick stained thin blood smear of *Babesia* from the giant panda. Final magnification of 1000×. *Scale-bars*: 5 μm

*Babesia*-like parasites were observed from in the blood smear of the giant panda under the magnification of ×1000 after staining with Giemsa and Diff Quick (Fig. 1). The form of parasite was round to oval (or ring-shaped) and elongated with a pointed end (Fig. 1). These single forms were usually present close to the center of erythrocytes. The nuclei appeared as rounded forms along the outer limits. In addition, we have found paired pyriform merozoites of *Babesia*, but no typical cruciform merozoites. The mean size of the merozoite was 1.97 ± 0.35 μm by 1.29 ± 0.11 μm (range 1.54–3.19 μm by 0.93–1.63 μm; *n* = 20).

### Detection of *Babesia* spp. by PCR

**Fig. 2:**
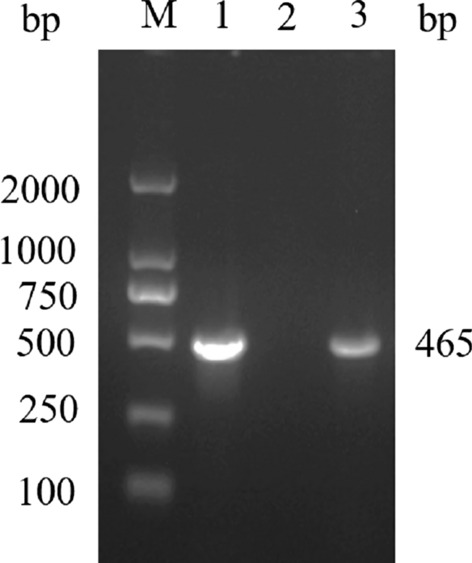
PCR of *Babesia* identification (465 bp). Lane M: Marker DL 2000 (Sangon Biotech^®^Shanghai, China); Lane 1: positive control (synthetic *B. canins* plasmid); Lane 2: negative control (nuclease-free water); Lane 3: the blood sample of the giant panda

PCR amplified the target sequence for *Babesia* species in the positive control, but the sample of nuclease-free water (negative control) did not show any bands. The collected sample from the giant panda was subjected to PCR. The results showed a 450 bp specific band which is consistent with the predicted size (Fig. 2). The amplified fragments were cloned and sequenced. Blast in NCBI demonstrated that the cloned sequence is most closed to a species of *Babesia* identified from bears (GenBank: AB566229.1).

### Amplification and sequencing of full length of *Babesia 18S* rRNA gene

**Fig. 3:**
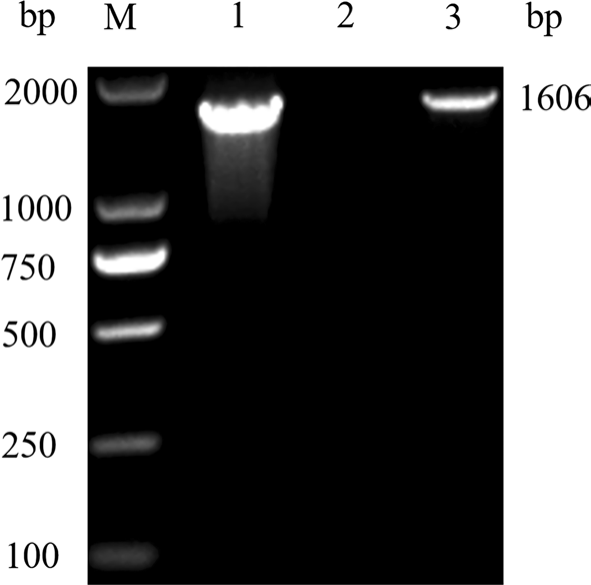
PCR of *Babesia* 18S rRNA (1600 bp) Lane M: Marker DL 2000 (Sangon Biotech^®^Shanghai, China); Lane 1: positive control (synthetic *B. canins* plasmid); Lane 2: negative control (nuclease-free water); Lane 3: the blood sample of the giant panda

One *Babesia* specific *18S* rRNA PCR band was successfully amplified (Fig. 3). An amplicon of 1604 bp comprising the majority of the *18S* rRNA gene was sequenced from the blood of the giant panda (GenBank: MT256300.1). The sequence of *Babesia 18S* rRNA from giant panda differed from other *Babesia* species, with the most similar species being *Babesia* sp*.* EBB (GenBank: AB566229.1) and *Babesia* sp. Iwate (GenBank: AB586027.1) which infect Japanese black and brown bears. The *Babesia 18S* rRNA from the panda showed 99.56% and 99.07% similarity and 7–8 bp difference compared with *Babesia* sp. EBB (GenBank: AB566229.1) and *Babesia* sp. Iwate (GenBank: AB586027.1), and a 93.15% similarity and 110 bp difference compared with *B. canis*; compared with *B. divergens*, there was 96.69% similarity and 53 bp difference (Table 1).

**Table 1:** Comparative results of the sequences of several *Babesia* and *Babesia* sp. EBP01

	No. of base-pair differences	Similarity (%)
*Babesia* sp. Iwate (AB586027.1)	7	99.56
*Babesia* sp. EBB (AB566229.1)	8	99.50
*Babesia* sp. Akita (AY190123.1)	15	99.07
*Babesia* sp.AJB-2006 (DQ028958.1)	16	99.00
*B. odocoilei* (KC4600321.1)	44	97.26
*B. divergens* (AB975389.1)	53	96.69
*B.gibsoni* (KC461261.1)	68	95.77
*B. caballi* (EU888904.1)	99	93.84
*B. orientalis* (AY596279.1)	109	93.21
*B. canis* (L19079.1)	110	93.15
*B. ovata* (AY081192)	127	92.09
*B. microti* (LC127372.1)	197	87.73
*B. bovis* (KF928959.1)	258	83.94

### Molecular phylogeny

**Fig. 4:**
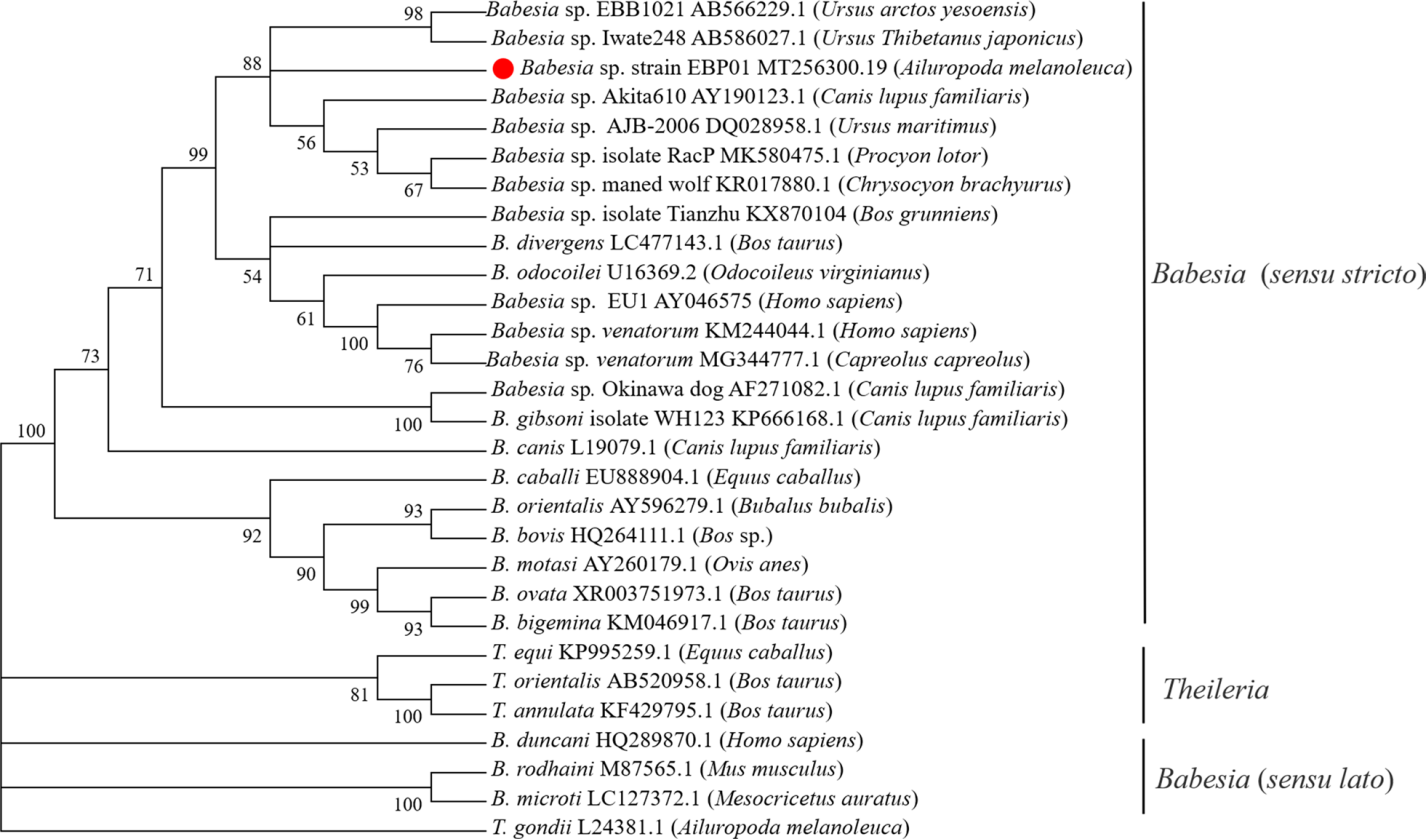
Rooted 50% majority-rule consensus cladogram of the open reading frame of the *18S* rRNA from piroplasms. Calculated by neighbor-joining and bootstrap estimates from 1000 replicates, with *T. gondii* as the outgroup. The bootstrap values based on 1000 replicates are displayed next to the branches, the hosts are listed in parentheses

The nucleotide sequences of the *18S* rRNA gene were used to reveal the phylogenetic relationship of the *Babesia* sp. from the giant panda with other *Babesia* species. The sequences identified in this study were aligned to known sequences of *18S* rRNA (1604 nt) of 29 *Babesia* spp. in the GenBank database. For the phylogenetic tree of the *18S* rRNA gene (Fig. 4), *T. gondii* (L24381.1) was used as the outgroup, *Babesia* strains formed two clades. One clade represented *Babesia* (*sensu lato*) and included *B. microti*, *B. rodhaini* and *B. duncani* which was close to the outgroup*.* The second clade represented *Babesia* (*sensu stricto*) and included *B. orientalis*, *B. canis*, *B. gibsoni* and other *Babesia* species which was far from the outgroup. The clade of Theileriidae was in the middle of *Babesia* (*s.s.*) and *Babesia* (*s.l.*). *Babesia* sp. detected in the giant panda is similar to the species which infects *Ursus thibetanus japonicus* (GenBank: AB586027.1) and *Ursus arctos yesoensis* (GenBank: AB566229.1) in Japan.

## Discussion

*Babesia* spp. are apicomplexans that live in erythrocytes similar to *Plasmodium* and *Theileria*, and have a wide host range, including hundreds of mammal species and a limited number of bird species [22–26]. *Babesia* parasites are maintained in a complex system of tick vectors and animal reservoirs [27]. Although there are many kinds of animals infected with *Babesia* spp., up to now, there is no report about the infection of *Babesia* and resulting babesiosis in the giant panda.

To our knowledge, this study is the first to confirm that the giant panda can be infected with *Babesia* spp. causing babesiosis. Usually, babesiosis in wildlife is mostly asymptomatic [28], but the panda in this study showed some clinical signs like fever, anemia, jaundice and hemoglobinuria. There are also some reports about babesiosis with clinical signs in wildlife. Hoby et al. [12] reported that five cases of fatal babesiosis in free-ranging chamois (*Rupicapra r. rupicapra*) attributed to infections with *Babesia capreoli*. Until now, we do not know which of the signs of babesiosis showed immediately after the giant panda was infected with *Babesia* or how the giant panda presented a chronic asymptomatic carrier state and then showed signs under specific circumstances. Further investigation is needed.

The common etiological diagnosis of babesiosis is established by the microscopic identification of the organism on stained blood smears [29]. However, if the number of parasites in the blood is low, it is difficult to detect them in the blood under the light microscope during the different stages of infection, and also detection is affected by the conditions and time of blood preservation. Therefore, a sensitive and specific diagnosis method is needed for early diagnosis of *Babesia* in the giant panda. A variety of immunological methods have been reported for the diagnosis of babesiosis in animals and humans [30]. However, due to the lack of specific giant panda antibodies against the disease, diagnostic methods still need to be developed. PCR amplification of DNA of *Babesia* is a more sensitive detection method, especially when *Babesia* infection is subclinical or parasitemia level is relatively low. Therefore, we used the combination of microscopy and PCR for the detection of *Babesia* infection in the giant panda [31].

Morphologically, *Babesia* is generally divided into two forms: large form (2.5–5.0 μm long) which include *B. bigemina*, *B. caballi* and *B. canis*, etc.; and small from (1.0–2.5 μm long) which include *B. bovis*, *B. gibsoni*, *B. microti*, *B. rodhaini*, etc. The orientation of the parasite in the red blood cells (RBCs) depends on its size, large pyriform parasites meet each other in an acute angle at the tips while small parasites make an obtuse angle to each other [32]. *Babesia* are polymorphic intraerythrocytic parasites and appear under light microscopy as ring forms, pear-shaped forms, paired pyriforms, and pleomorphic ring forms [33]. In this study, under a microscope, we found round to ring-shaped, paired pyriforms and other shapes indicating *Babesia*, but no typical cruciform merozoites. According to aforementioned size of the *Babesia* infecting the giant panda should belong to the group of small *Babesia*, but the sample is from just one panda, therefore we need more data to prove it.

We used *18S* rRNA gene of *Babesia* as a conserved identification sequence. In addition, *18S* rRNA gene is widely used in *Babesia* research, making it easier to compare this undetermined species with other known *Babesia* species. The *18S* rRNA gene, with conservative regions provide a sufficient template DNA for PCR, and is widely used as a target for *Babesia* species identification. Although it has been reported that there is a high degree of conservation of the *18S* rRNA sequences in *Babesia* and *Theileria* species, it has been suggested to use the full *18S* rRNA gene, especially when dealing with new organisms, to ensure that genetic variation is not overlooked [34].

To our knowledge, our study is the first to provide information of a *Babesia* species infecting the giant panda. This *Babesia* sp. is likely to be a novel species based on the phylogenetic analysis which has been widely used to study the genetic diversity of *Babesia* [35–37]. This clearly suggests that this novel *Babesia* sp*.* is separated from the other types. The phylogenetic tree (Fig. 4) showed that *B. odocoilei* and *B. divergens* are highly similar with the *Babesia* sp. strain EBP01. It is important to note that *B. divergens* will cause amphixenosis and has the risk of infecting humans [38, 39].

Nonetheless, *Babesia* sp. strain EBP01 is closely related with *B. odocoilei* and *B. divergens* which is closely linked to other undetermined *Babesia* species which were found in both the black and brown bear, dog, raccon and wolf [40, 41]. They formed a distinct clade which was separated from the clade consisting of *B. divergens* and *B. odocoilei*. These clades are clearly separated with a bootstrap value of 99% (Fig. 4). These *Babesia* species have about 99.7% similar gene sequences associated with the *Babesia* spp. infecting the bears and are significantly different from several common species of *Babesia* such as *B. divergens*, *B. canis*, *B. bovis*, *B. ovata* and *B. gibsoni* (Table 1). It is hypothesized that the *Babesia* sp. strain EBP01 is possibly a novel *Babesia* species unique to *A. melanoleuca*, which is close to the species of *Babesia* which infect other ursids; however, we need more evidence and investigation to verify this.

The threat of domestic dogs to biodiversity conservation is increasingly addressed in the literature, but is often overlooked and underestimated in conservation programmes worldwide [42, 43]. It is well known that domestic dogs and giant pandas share many common diseases, such as canine distemper, parvovirus and parasitic infections. As a result, it is very important to carry out and strengthen management of free-ranging dogs around the nature reserves to better protect local wildlife, such as giant pandas [44, 45]. Interesting, our results showed that a *Babesia* species Akita610, collected from dogs in Japan, is closely related to the *Babesia* sp. strain EBP01 (99.07%). Although the above findings are not sufficient to indicate that dogs may also be infected with *Babesia* sp*.* strain EBP01, this result will help us try to investigate the *Babesia* infection in sympatric species of the giant panda, especially canids*.*

*Babesia* spp. are tick-borne parasites, thus it is important to find the specific definitive tick host, for the continued study of *Babesia* sp. in the giant panda [46, 47]. It is worth noting that captive giant pandas have a history of tick infestation. Obviously, ticks are the main threat of babesiosis in the giant panda; however, the specific species of tick that infect giant pandas and transmit *Babesia* need further study.

## Conclusions

To our knowledge, this study found the first case of *Babesia* infection in the giant panda. We successfully detected the pathogen of *Babesia* in the blood from a giant panda by microscopic examination and PCR method, and found evidence using molecular biology to show the *Babesia* sp*.* is more closely linked to unidentified *Babesia* species found in bears in Japan. We think that this *Babesia* sp*.* may be a novel species, currently named *Babesia* sp*.* strain EBP01.

## Data Availability

All data generated or analyzed during this study are included in this published article. The newly generated sequence was deposited in the GenBank database under the accession number MT256300.1.

## Abbreviations

PCR: Polymerase chain reaction
18S rRNA: 18 Svedberg ribosomal RNA
MODS: Multiple organ dysfunction syndrome
IUCN: International Union for Conservation of Nature
EDTA: Ethylene diamine tetraacetic acid
